# Does Endometrial Thickness or Compaction Impact the Success of Frozen Embryo Transfer? A Cohort Study Analysis

**DOI:** 10.3390/jcm13237254

**Published:** 2024-11-28

**Authors:** Nardin Aslih, Yuval Atzmon, Asaf Bilgory, Yasmin Shibli Abu Raya, Moamina Sharqawi, Einat Shalom-Paz

**Affiliations:** IVF Unit, Hillel Yaffe Medical Center, Hadera 3820302, Israel; atzmony@gmail.com (Y.A.); asaf_bil@hotmail.com (A.B.); yasmineshibli@gmail.com (Y.S.A.R.); mo2mena-9@hotmail.com (M.S.); einatshalompaz@gmail.com (E.S.-P.)

**Keywords:** IVF, FET, natural cycle, endometrial thickness, endometrial compaction

## Abstract

**Background**: In frozen embryo transfer (FET) cycles, optimal endometrial thickness on ovulation day is typically 7–8 mm before progesterone administration. Recent studies have highlighted the significant inverse correlation between ongoing pregnancy rates and changes in endometrial thickness during the secretory phase after progesterone exposure, particularly by the day of embryo transfer (ET). This study aims to investigate how changes in endometrial thickness from the end of the proliferative phase to ET impact FET outcomes. **Methods**: We conducted a prospective observational cohort study involving 247 FET cycles, divided into hormonally based (102) and ovulatory-based (145) groups. Patients were monitored through transvaginal ultrasound (TVS) and blood tests. On ET day, we assessed endometrial thickness and changes compared to the last day of the proliferative phase, defining endometrial compaction as a decrease in thickness. We analyzed data to identify factors predicting cycle outcomes. **Results**: The study reported chemical and clinical pregnancy rates of 47.4% and 38.1%, respectively. Endometrial compaction was observed in 37.2% (92/247) of cycles, with corresponding rates of 48.9%, 32.6%, and 29.5% for chemical, clinical, and ongoing pregnancies, compared to 46.4%, 41.3%, and 28.9% in cycles without compaction. These differences were not statistically significant, and patterns of endometrial thickness change were similar across different FET protocols and progesterone exposure durations. **Conclusions**: The main factors influencing cycle outcomes were maternal age, embryo transfer protocol, and endometrial thickness on ET day, with endometrial compaction showing no correlation with improved outcomes.

## 1. Introduction

Since the advent of vitrification, frozen embryo transfer (FET) cycles have steadily increased worldwide [1,2]. With more frozen embryos available for future use, there has been a rise in the cumulative live birth rate per oocyte retrieval cycle [1,3].

Several factors have contributed to the rise in frozen embryo transfers (FETs), starting with advancements in incubators and a shift towards vitrification for embryo preservation. This change has enhanced the survival rates and quality of thawed embryos [3] supported by reassuring safety data [4,5]. The reasons for embryo freezing have expanded beyond concerns of ovarian hyperstimulation syndrome to include planned embryo freezing for future use, pre-gestational testing, premature progesterone rise in the late follicular phase, and suboptimal endometrial conditions. These factors all contribute to the increase in FET cycles and help achieve optimal results in assisted reproductive technology (ART) [6,7].

As the trend towards more cryopreservation of embryos is still on the rise, the question that remains is the following: what is the most important factor contributing to FET success? Is it the endometrial preparation protocol? Is it the timing of the embryo transfer? Is it the endometrial thickness or perhaps the endometrial microscopic structure?

Two main protocols for endometrial preparation in FET cycles are currently in use: artificial and natural. Previous retrospective publications report contradicting data regarding the superiority of one protocol over the other, with a trend towards a preference for natural cycles; well-designed randomized controlled trials are still lacking [8,9,10,11,12,13].

Both FET protocols are based on primary endometrial exposure to either endogenous or exogenous estrogen and later exposure to progesterone (either endogenous following ovulation or exogenous administered in various routes). Protocols vary regarding the dose and timing of exposure to estrogen in artificial protocols; whether to induce ovulation and, if so, the timing of the induction; and the timing of embryo transfer after endometrial exposure to progesterone [14,15,16].

Monitoring during FET cycles involves repeated ultrasound (US) examinations to assess the endometrium and the development of leading follicles and, if needed, blood tests for hormone levels. Combining this information during follow-up helps clinicians determine the timing of embryo transfer.

The endometrium is a complex tissue composed of multiple cell types that undergoes hormone-influenced dynamic remodeling to establish a microenvironment to support the implanting embryo [17].

Decidualization is the transfer from the proliferative phase to the secretory phase which involves significant morphological and functional changes in the stromal cells. For successful implantation, precise timing and coordination are essential to establish the “implantation window”. Some authors assumed that these changes are expressed as endometrial compaction in US scans and can be monitored and visualized using ultrasound [18,19].

There is wide agreement in the literature that a minimum endometrial thickness of 6–7 mm at the end of the follicular phase is necessary for optimizing cycle results [20,21,22]. Only a few studies investigated sonographic changes in the endometrium during the luteal phase. Those studies disagreed on the association between these changes and cycle results [18,19,23,24,25,26,27,28,29,30,31,32,33].

The aim of this study was to explore whether changes in endometrial thickness during the luteal phase correlate with the results of FET cycles of the cleavage stage and blastocyst transfer.

## 2. Materials and Methods

### 2.1. Patients

This prospective observational cohort study was conducted in a single reproductive center, Hillel Yaffe Medical Center—Hadera, Israel, from 1 January 2021 to 31 July 2023.

Exclusion criteria were refusal to provide informed consent, known uterine anomaly (for example, unicornuate or bicornuate uterus), intramural fibroids > 3 cm, submucosal fibroids, endometrial polyps of any size, known endometrial pathology (for example, consistently thin endometrium throughout treatments (<6 mm in the end of the follicular stage)), premature progesterone elevation, use of donor oocytes, maternal age > 43, and repeated implantation failure (defined as ≥3 consecutive unsuccessful transfers).

Records of all patients and their cryopreserved embryos were analyzed. Data collection included baseline parameters of age, body mass index (BMI; kg/m^2^), type of infertility, FSH levels (as an indicator of patients’ ovarian reserve), parity, lifestyle, and cause of infertility. Treatment parameters including number of embryos transferred, embryo quality, and pregnancy outcomes were also evaluated.

Institutional Review Board approval was obtained. All patients signed informed consent forms.

### 2.2. Treatment Protocol

All patients scheduled for FET underwent their first ultrasound evaluation during the early follicular phase. At that point, the physician would decide whether to proceed with artificial frozen embryo transfer (aFET) or ovulatory cycle frozen embryo transfer (ovu-FET). The choice of protocol and medication was determined by the patient’s medical history, previous cycles, and, when possible, their preferences.

### 2.3. Artificial FET Protocol (aFET)

Estradiol 2 mg (Estrofem^®^ Novo Nordisk, Kalundborg, Denmark), taken three times a day (TID), was initiated following the first visit in the early follicular phase (days 3–5 of the menstrual cycle) and continued for at least 8 days. On the 8th day of treatment, the patient returned for a second visit, where the endometrium was evaluated using transvaginal ultrasound (TVS). If the endometrial thickness exceeded 8 mm, progesterone treatment was started. Progesterone options included oral dydrogesterone 10 mg TID (Duphastone^®^ Abbott, Tokyo, Japan), vaginal micronized progesterone (MVP) 100 mg TID (Endometrin^®^, Ferring, Hong Kong), or MVP gel 90 mg twice a day (BID) (Crinone^®^ 8%, Merck Serono, Darmstadt, Germany). The duration of progesterone administration prior to embryo transfer depended on the embryo’s stage: cleavage stage embryos were transferred after 4 days of progesterone, while blastocysts were transferred after 6 days [15].

### 2.4. Ovulatory Cycle Protocols (Ovu-FET)

In ovulation-based cycles, ovulation was either natural or induced with Letrozole. Further on, ovulation was either natural or triggered with 250 mcg of recombinant hCG (Ovitrelle^®^; Merck-Serono) when the leading follicle was 18 mm and endometrial thickness was more than 7.5 mm, as seen on TVS.

In Letrozole-induced cycles, patients were treated with 2.5 mg of Letrozole (Letrozole^®^, Teva, Israel), BID for 5 days, from day 5 to day 9 of menstruation. TVS monitoring continued until a dominant follicle reached 18 mm and the endometrial thickness exceeded 7.5 mm.

In all cases, the decision to proceed with a fully natural cycle with spontaneous ovulation or to trigger ovulation with Ovitrelle^®^ was made based on the timing of the transfer, with efforts made to avoid weekend transfers.

### 2.5. Patient Monitoring

Patients were followed during the treatment with TVS and blood tests (serum Estradiol, Progesterone, and LH levels), as necessary. The dating of embryo transfer was scheduled based on the embryo’s age and days of progesterone exposure.

On the day of the FET, TVS was performed to assess the endometrial thickness and pattern of change prior to transfer. All US images were recorded and saved.

Endometrial compaction was defined as a reduction in endometrial thickness, documented using TVS on the day of transfer, compared to endometrial thickness on the last day of the proliferative phase.

### 2.6. Pregnancy Determination

Chemical pregnancy was diagnosed when β-hCG levels exceeded 50 mIU/mL 12 days after embryo transfer. A clinical pregnancy was confirmed if a gestational sac with a detectable fetal heartbeat was observed on ultrasound at 6 weeks of gestation. An ongoing pregnancy was defined as the presence of fetal cardiac activity on transvaginal ultrasound (TVS) at 12 weeks or later, or a live birth. Demographic data, treatment details, pregnancy follow-up, and outcomes were all recorded.

### 2.7. Statistical Analysis

All study parameters were evaluated using descriptive statistics of mean, standard deviation, median, percentage, and range. Differences between the aFET and ovu-FET protocols as continuous parameters were evaluated using *t*-test or Mann–Whitney U test and categorical parameter with Fisher exact test or Pearson chi square. Receiver Operating Characteristic (ROC) curves with sensitivity and specificity values were used to find the best cutoff for endometrial thickness to predict a clinical pregnancy. A multivariate logistic regression model was used to predict clinical pregnancy, with adjustment for several independent parameters. *p* < 0.05 was considered significant. SPSS version 28 was used for all statistical analyses.

## 3. Results

A total of 664 FET cycles were conducted in our unit during the abovementioned period, of which 247 FET cycles were included in this study. Patients’ demographics and characteristics are presented in Table 1.

Chemical and clinical pregnancy rates in the study group were 47.4% and 38.1%, respectively.

As presented in Table 2, endometrial thickness was not affected by the treatment protocol. However, endometrial compaction (negative delta) and clinical and ongoing pregnancy rates were significantly higher in the ovulatory-based FET cycles. Median compaction was 0.11 mm and 0.12 mm in both groups, which is not statistically significant.

Endometrial compaction occurred in 92 of the 247 FET cycles included in the study (37.2%), 29/102 (28.4%) in the aFET group and 63/145 (43.4%) in the ovu-FET cycles. No statistical difference was observed in cycle outcomes regarding the occurrence of endometrial compaction (Table 3).

We found that the rates of compaction between groups, according to the day of transfer (reflecting length of progesterone exposure), were similar. In transfers of cleavage stage embryos, compaction occurred in 38.4% of cycles, while the rate of compaction in blastocyte transfers was 35.4% (two additional days of progesterone exposure). Pregnancy rates among groups were comparable.

Multivariant analysis was performed to identify factors affecting pregnancy rates. Treatment protocol, maternal age at transfer, and endometrial thickness (and not endometrial compaction) were found to be successful predictors of clinical pregnancy.

Using ROC analysis, we attempted to identify the endometrial thickness and change in thickness which can be used to predict clinical pregnancy. When endometrial thickness was more than 8.45 mm on the day of embryo transfer, clinical pregnancy rates were 52.7%, compared to 36.6% when it was less than 8.45 mm (*p* = 0.021).

An endometrial thickness of 8.45 mm was found to predict clinical pregnancy with a sensitivity of 79.8% and specificity of 41.2%.

Unfortunately, this analysis did not identify a change in endometrial thickness (compaction) that could be used to predict cycle outcomes.

## 4. Discussion

This prospective cohort study evaluated the impact of endometrial compaction in ovulatory cycle and artificial cycle FET protocols. The endometrium was measured on the day before progesterone exposure and repeated on the day of transfer using TVS. The analysis was categorized according to cleavage stage and blastocyst transfers.

We found endometrial compaction (decrease in endometrial thickness) in only 37.2% of the FET cycles, ranging from 0.06 to 0.17 mm in both groups. However, multivariant analysis did not demonstrate a significant impact of the compaction on treatment outcomes.

Key factors affecting cycle outcomes were maternal age on the day of ovum pick-up, the protocol used for ET, and final endometrial thickness on the transfer day. Importantly, our results support the growing evidence in the literature of significantly better cycle results with ovu-FET compared to aFET.

Despite advances in the field of assisted reproduction, overall pregnancy and implantation rates have remained relatively low. Extensive research has been undertaken to improve success rates.

In light of the substantial rise in the number of FET cycles performed, a great deal of interest has been directed towards optimizing the results. The endometrium is one of the most studied components. It is well known that the endometrium undergoes two stages throughout the menstrual cycle: proliferative (follicular) and secretory (luteal). During the proliferative phase of the cycle, estrogen levels increase, stimulating angiogenesis and the growth of endometrial glands. This results in thickening of the endometrium and the characteristic tri-laminar appearance seen on ultrasonography [19,34,35].

Following ovulation, elevated progesterone levels alter the endometrium, reducing proliferation while promoting more intense growth of glands and blood vessels. Glycogen accumulates in the glandular lumens, causing the glands and vessels to become more tortuous [34,36].

Endometrial receptivity refers to the endometrium’s ability to successfully attach the embryo, support its growth, and sustain its viability. This is achieved only after the endometrium undergoes the abovementioned changes and increases in thickness. However, a thorough understanding of the biological and histological interactions between the endometrium and embryo has yet to be applied clinically.

Histological changes can only be assessed through biopsy, whereas transvaginal ultrasound (TVS) offers a non-invasive, simple, and reliable method to measure parameters such as thickness, volume, and pattern. These measurements can serve as indirect indicators of the endometrium’s receptive quality.

The shift towards the secretory phase is characterized by increasing echogenicity of the endometrial lining, demonstrated on ultrasound while its thickness increases minimally [35]. These changes may be interpreted as compaction measured on the ultrasound exam.

Some authors suggested that lack of endometrial compaction may be due to the presence of progesterone receptor deficiency or endometrial resistance among some infertile women [19].

As mentioned, only a few studies investigated the sonographic changes in the endometrium during the luteal phase. Those studies disagree on the association between the changes and cycle results [18,19,23,24,25,26,27,28,29,30,31,32,33].

The contradicting results reported by various groups may arise from different methodologies. One factor could be the sonographic mode of measuring the endometrial lining; while some groups used TVS, others used transabdominal US (TAS).

Since the introduction of US to the field of medicine by Dr. John J. Wild in 1951, many advances have occurred in the field of US imaging. Due to higher-frequency probes and the proximity of the TVS probes to the pelvic organs, TVS has become the standard tool used for patient monitoring during IVF cycles. TVS overcomes the two disadvantages to the use of transabdominal US: the requirement for a full urinary bladder, which often causes discomfort, and image quality being seriously compromised in obese patients [37]. Studies emphasize the superiority of TVS over TAS in assessing endometrial thickness accurately [38].

Moreover, Olgan et al. demonstrated a higher rate of endometrial compaction when measured by TAS compared to TVS in the same patients, suggesting inaccuracy of endometrial assessment and thickness measurement by TAS [28]. A positive correlation was found between BMI and endometrial thickness change measured by TAS. Thus, obesity might be a confounding factor. Obesity interferes with TAS because it is difficult for ultrasound wave signals to penetrate fat to picture the organs underneath [39].

Endometrial compaction is defined as the decrease in endometrial thickness from the end of the follicular phase to the day of embryo transfer (ET) as measured by ultrasound [18,19,40]. Some reports emphasized the importance of endometrial compaction on pregnancy rates and live birth rates. Two retrospective, observational studies from a single infertility center found a positive correlation between endometrial compaction and ongoing pregnancy rates in hormonally prepared FET cycles [18,19].

However, following those promising results, several studies on the predictive value of endometrial compaction for live birth rates or ongoing pregnancy rates presented conflicting results [28,29,30,31,32,33].

Two recent reviews and meta-analyses concluded that the predictive value of endometrial compaction in determining the live birth rate and other cycle outcomes is limited, and assessment of endometrial compaction may no longer be necessary [27,40]. Our results are consistent with these publications and question once more the need for evaluating endometrial thickness on the day of transfer and, even more so, making any clinical decisions according to it.

Based on our data, we noticed another intriguing observation: that longer exposure to progesterone did not lead to higher compaction rates.

When analyzing the data by the day of transfer (cleavage stage embryo vs. blastocyte transfer), endometrial compaction rates were found to be similar when the ET was performed on day 2, 3, or 5 of the luteal phase, as were pregnancy rates. This observation questions the assumption that endometrial compaction adequately reflects the effect of progesterone on the endometrium.

This prospective observational study focused on the endometrium as a major factor contributing to cycle results and specifically on the finding of endometrial compaction as a possible factor affecting success rates.

TVS and blood hormone levels were used to monitor patients undergoing FET cycles with different protocols. The results did not indicate any significant relation between endometrial compaction and an effect on chemical, clinical, or ongoing pregnancy rates. Other factors affecting success rates were, unsurprisingly, maternal age and endometrial thickness on the last day of transfer.

Interestingly, this study demonstrated significantly higher clinical and ongoing pregnancy rates in the ovulation-based cycles compared to the artificial-based cycles. This finding contributes to the growing evidence in the literature, including a previous report by our group [41,42].

The advantages of the present study are that it is a prospective study that evaluated ovulatory-based and artificial FET cycles separately. All patients were examined using TVS for endometrial assessment, which is known to be superior to TAS. A limited number of US technicians performed the US exams to minimize interobserver variations.

Limitations of the study include that different embryos of cleavage stage and blastocyst were transferred and we did not know the genetic status of the embryos, since preimplantation testing is not commonly performed in Israel.

## 5. Conclusions

Several key factors influence the outcome of embryo transfer cycles, including maternal age, the specific protocol used for the embryo transfer, and the measurement of endometrial thickness on the day of transfer. One noteworthy observation is the phenomenon of endometrial compaction, which has been the subject of various theories attempting to explain its occurrence. However, despite ongoing research and the accumulation of data, there is no clear evidence to suggest that endometrial compaction plays a significant role in determining the success or failure of treatment outcomes.

## Figures and Tables

**Table 1:** Patient demographics and baseline characteristics.

Variable	Value
Age of patient at transfer	33.6 ± 6.4 years
Age of patient at embryo freezing	32.3 ± 6.5 years
Body mass index	26.7 ± 5.9 kg/m^2^
Smoker	49 (20%)
Basal FSH	7.56 ± 3.93 IU/L
**Cause of infertility**	
Unexplained	61 (25%)
Male	87 (35%)
Anovulation	48 (19%)
Mechanical	45 (18%)
Advanced maternal age (>40 years)	26 (10.5%)
**Parity**	
0	127 (51%)
1	97 (39%)
2	19 (8%)
3 + 4	4 (2%)
Number of transfers	3.85 ± 2.5
Cleavage embryo transfers	152 (61.1%)
Blastocyst transfers	96 (38.9%)
**Number of embryos transferred**	
1	214 (87%)
2	32 (13%)
3	1 (0.4%)

**Table 2:** Cycle characteristics and pregnancy outcomes.

Variable *	Artificial FET n = 102	Ovulatory FET n = 145	*p*-Value
Endometrial thickness at the end of follicular phase (mm)	8.87 ± 1.5	9.04 ± 1.7	0.43
Endometrial thickness at the day of transfer (mm)	9.72 ± 1.98	9.62 ± 2.33	0.72
Endometrial compaction (negative delta) n (%)	29/102 (28.4%)	63/145 (43.4%)	**0.016**
Endometrial compaction (mm—median)	−0.11 [(−0.17)–(−0.06)]	−0.12 [(−0.16)–(−0.07)]	0.59
Chemical pregnancy	42/102 (41.1%)	75/145 (51.7%)	0.10
Clinical pregnancy	29/102 (28.4%)	65/145 (44.8%)	**0.009**
Ongoing pregnancy	16/101 (15.9%)	55/142 (38.7%)	**<0.001**

* Data are presented as mean ± standard deviation or n (%). Values statically significant in bold format.

**Table 3:** Endometrial compaction did not affect cycle outcomes.

Type of Pregnancy	Negative Delta— Compaction (n = 92)	Zero or Positive Delta— No Compaction (n = 155)	*p* Value
Chemical	45/92 (48.9%)	72/155 (46.4%)	*p* = 0.71
Clinical	30/92 (32.6%)	64/155 (41.3%)	*p* = 0.17
Ongoing *	27/91 (29.7%)	44/152 (28.9%)	*p* = 0.90

* Missing data—4 cases.

## Data Availability

The data presented in this study are available on request from the corresponding author. The data is not publicly available due to privacy.
